# Assessing Pain Linked to High Metabolic and Glycolytic Shift in Neurofibromatosis Type 1 (NF1) Patients by Using a Bioenergetic Biomarker: A Pilot Study

**DOI:** 10.7759/cureus.111113

**Published:** 2026-06-18

**Authors:** Grover P Miller, Kimberly Krager, Dayoung Eom, Nukhet Aykin-Burns, Erika Santos-Horta

**Affiliations:** 1 Biochemistry and Molecular Biology, University of Arkansas for Medical Sciences, Little Rock, USA; 2 Pharmaceutical Sciences, University of Arkansas for Medical Sciences, Little Rock, USA; 3 Neuro-Oncology, University of Oklahoma Health Sciences Center, Oklahoma City, USA

**Keywords:** bioenergetics, glycolysis, mitochondria, neurofibromatosis, nf1, pain, peripheral blood mononuclear cells, pilot, prospective observational study

## Abstract

Background and objective

Neurofibromatosis type 1 (NF1) is a common, incurable genetic disorder that causes neuronal tumors and other symptoms. Emerging evidence suggests that neurofibromin mutations dysregulate mitochondrial metabolism. We developed and tested a biomarker approach for the first time to assess mitochondrial dysfunction among NF1 patients and its association with adverse health outcomes.

Methods

We conducted a prospective observational clinical study, enrolling 44 NF1 patients and 11 controls without neurofibromin defects. We isolated peripheral blood mononuclear cells (PBMCs) from the subjects and measured mitochondrial function. Patient data were collected from biomedical evaluations and questionnaires assessing fatigue and pain; these results were then correlated with mitochondrial measurements.

Results

NF1 led to significant differences in the extracellular acidification rate (ECAR) (p = 0.008) but not in oxygen consumption rates (OCR). The OCR versus ECAR plot indicated a glycolytic bias in NF1 patients. Patient-reported pain was positively correlated with OCR (p = 0.003) but not with ECAR (p = 0.4). There were no correlations observed for fatigue. The severity of pain and fatigue were also positively correlated (p < 0.002).

Conclusions

This pilot project was the first study in humans to suggest that NF1 patients may have a dysregulation in cellular respiration towards less aerobic, more glycolytic metabolism. Additionally, the findings suggest that the pain experienced by NF1 patients may be linked to this energy dysfunction.

## Introduction

Neurofibromatosis type 1 (NF1) is a common, yet incurable, genetic disorder affecting approximately one in 2,750 people worldwide [1,2]. Its hallmark traits include various types of peripheral and central nervous system tumors, along with a wide range of symptoms such as pigmentary lesions, cognitive deficits, and musculoskeletal abnormalities [2,3]. The clinical phenotype arises from predominantly point mutations (90%) in the NF1 tumor suppressor gene, which encodes neurofibromin [4]. The most well-characterized function of neurofibromin is its stimulation of Ras GTPase activity, leading to Ras inactivation and suppression of signaling through multiple effectors, including mTOR, MEK, ERK, and, indirectly, cAMP/PKA [5-7]. The wide range of NF1 mutations and hereditary mosaicism pose significant challenges for understanding the relationship between genotype and phenotype. This variability is also observed in the clinic, where even monozygotic twins can present with different medical complications, highlighting the potential importance of epigenetics in NF1 syndrome [8,9].

Emerging evidence suggests that neurofibromin mutations dysregulate both cellular and organismal metabolism. NF1 patients exhibit decreased respiratory quotients (the ratio of carbon dioxide eliminated per unit of oxygen consumed), indicating that neurofibromin defects alter their basal metabolic rates [10]. Similarly, animal knockout models demonstrate reduced respiratory quotients, decreased lipid storage, and increased energy expenditure [11-13]. Further analysis of isolated cells lacking neurofibromin reveals lower basal and coupled respiration, diminished maximal and spare respiratory capacities, and elevated levels of reactive oxygen species. This emphasizes the critical role of neurofibromin in maintaining normal mitochondrial function [13].

Along with NF1 genetics, therapeutic interventions may also influence mitochondrial function. MEK inhibitors are the only FDA-approved treatment for plexiform neurofibromas in NF1 patients, yet these drugs are associated with high incidences of drug-induced toxicities [14-16]. Possible contributors to these adverse effects include their capacity to increase oxygen consumption rates and alter mitochondrial metabolism, affecting complex I and the electron transport chain [17,18]. Although there is no consensus in the field, vitamin D administration may potentially improve mitochondrial function, offering a basis for better health outcomes in patients [19].

In light of this information, we hypothesized that NF1 mutations may affect cellular bioenergetics in patients, potentially contributing to adverse health outcomes such as pain and fatigue. Both symptoms are commonly observed in patients with mitochondrial diseases and are particularly prevalent in individuals with NF1, to the extent that they are included as measured outcomes in clinical trials for this population [14-16,20].

## Materials and methods

Study design

This pilot study was conducted at the University of Arkansas for Medical Sciences (UAMS) from August 2023 to August 2024 with the following objectives: to investigate mitochondrial respiratory dysfunction in peripheral blood mononuclear cells (PBMCs) in patients with NF1; to investigate whether mitochondrial respiration dysfunction correlates with clinical symptoms; and to assess the impact of therapeutical interventions on mitochondrial function and metabolic plasticity in the circulating cells of NF1 patients. Subjects were recruited from the Adult Neurofibromatosis Clinic on campus, and consent was obtained before inclusion and testing. This study was approved by the UAMS Institutional Review Board (study number: 274877).

Subject population

The study included adult participants divided into two groups: a control group (comprising NF1 patients' chaperones) and an NF1 patient group. The eligibility criteria for the control group were being over 18 years of age and consenting to provide a 10 ml blood sample. The exclusion criteria for the control group included a personal or family history of neurofibromatosis or schwannomatosis. For the NF1 patient group, the eligibility criteria were adults over 18 years of age, a confirmed diagnosis of NF1, and agreement to provide serial blood samples as well as complete pain and fatigue questionnaires on the same day as blood collection. The exclusion criterion for the patient group was current treatment for a malignancy.

Clinical evaluation

During this study, we collected basic demographic information from NF1 patients and controls. NF1 subjects were active patients at the UAMS Adult NF Clinic, so we also collected additional data from standard biochemical laboratory tests, as well as BMI, height, and echocardiograms. These tests were performed in accordance with guidelines from the Mitochondrial Disease Society for evaluating patients being investigated for mitochondrial disorders [20]. The rationale for following this protocol was that this pilot study focuses on assessing energetic metabolism, and patients with mitochondrial diseases and NF1 patients share multiple clinical features, including short stature, fatigue, pain, and hearing disorders [20].

We collected a single blood sample from control subjects at the time of consent. For NF1 patients who consented, blood collections were performed three times at one-month intervals. This schedule was chosen because this pilot project aimed to explore how results might vary over time. It was also important to evaluate whether changes in oxygen consumption rates (OCR) and extracellular acidification rate (ECAR) correlated with clinical symptoms such as pain and fatigue, as well as with the use of MEK inhibitors. Additionally, pain and fatigue intensity in patients were assessed at each blood collection using the self-reported Numerical Pain Rating Scale (NRS-11) and the Functional Assessment of Chronic Illness Therapy-Fatigue (FACIT-F) scale.

Mitochondrial respiration and cellular bioenergetics

Mitochondrial function and cellular bioenergetics were measured in PBMCs isolated from collected blood samples. SepMate™ PBMC isolation tubes and Lymphoprep™ density gradient medium (STEMCELL Technologies, Vancouver, Canada) were used for PBMC isolation according to the manufacturer’s protocol. Freshly isolated cells were counted, and 225,000 cells per well were plated on Cell-Tak™ (Corning, Corning, NY) coated XF-Pro well plates using Seahorse XF DMEM assay medium supplemented with glutamine and pyruvate. The plate was centrifuged at 200 × g at room temperature for 90 seconds (acceleration = 1; deceleration = 0). After confirming cell attachment, the cells were incubated in a non-CO₂ incubator for 20 minutes at 37 °C.

The cellular bioenergetics of PBMCs were measured using an Agilent Seahorse XF-Pro Extracellular Flux Analyzer (Agilent, Santa Clara, CA). Specifically, we performed the standard mitochondrial stress test by consecutively injecting oligomycin (ATP synthase inhibitor, 10 μM), BAM15 (uncoupler, 3 μM; *N5,N6-Bis(2-fluorophenyl)(2,1,3)oxadiazolo(4,5-b)pyrazine-5,6-diamine)*, and the mitochondrial electron transport chain inhibitors antimycin A and rotenone (10 μM) into the wells, as previously described [21-23]. Technical replicates (a minimum of eight wells per condition) were run for each sample, and wells exhibiting injection artifacts were excluded. The resulting data were normalized to viable cell number, determined by trypan blue exclusion, in accordance with established PBMC extracellular flux protocols [24-26].

Statistical analyses**

All calculations were performed using the statistical package in GraphPad Prism version 10.5 (GraphPad Software, Boston, MA). Data were expressed as mean ± standard deviation (SD), with corresponding median values also reported. Descriptive statistics were presented as frequencies (percentages). Comparisons between two groups were made using an independent-sample t-test for normally distributed numeric variables. The Kolmogorov-Smirnov test was used to assess normality. Given differences in sample size between controls and NF1 patients, Welch’s t-test was applied when appropriate. Categorical data were analyzed using the Chi-square test. Associations between measured mitochondrial function tests (OCR and ECAR), reported clinical symptoms (fatigue and pain), patient characteristics (height and BMI), or vitamin D levels among NF1 patients were assessed using the Pearson correlation test. The potential association between patient-reported pain and fatigue was also evaluated using Pearson correlation. Statistical significance was set at p < 0.05.

## Results

Subject characteristics

**Figure 1:**
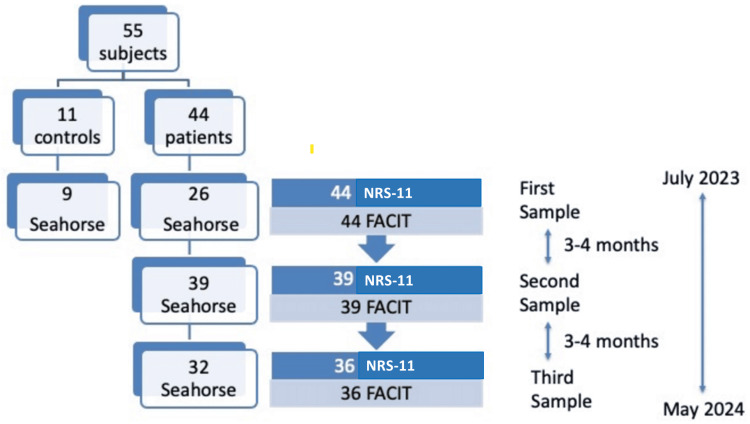
Study design illustration Group allocation (controls and NF1 patients) and breakdown for the number of subjects providing blood samples for Seahorse analyses and questionnaire responses for patient-reported fatigue (FACIT-F) and pain (NRS-11). When possible, NF1 patients provided samples and responses at each of the three visits, while controls provided only blood samples NF1: neurofibromatosis type 1; FACIT-F: the Functional Assessment of Chronic Illness Therapy-Fatigue; NRS: Numerical Pain Rating Scale

Forty-four patients with NF1 and eleven controls were enrolled in this study (Figure 1, Table 1). The NF1 patient group had an average age of 43 years, which was similar (p = 0.43, t-test) to that of the control group (48 years). The gender distribution varied slightly between the two groups but was not statistically significant (p = 0.12, Chi-square test). Anthropometric characteristics of the NF1 group indicated that half of the patients fell within the first percentile of the American adult population height distribution for their respective sexes. Only two patients (5%) had a BMI of less than 20. The biochemical profile revealed that the most common abnormality was low creatine kinase levels. Additionally, one quarter of the patients exhibited vitamin D deficiency at the time of consent or in the past. Similarly, one quarter of the patients had hemoglobin levels outside the normal range. Liver dysfunction was rare. A total of 31 patients underwent echocardiograms, with most showing normal results. Identified abnormalities included right-sided dilatation (1), interatrial septum abnormalities (1), structural valvular alterations (2), and pericardial effusion (1). Notably, one patient who had a normal echocardiogram at the beginning of the study experienced a 10% decrease in ejection fraction during treatment with a MEK inhibitor.

**Table 1:** Summary of the demographic characteristics of patients and controls ^1^Defined as height < 68 inches for males and < 64 inches for females. ^2^Normal range for creatinine kinase (CK): 50-289 U/L. ^3^Defined as a vitamin D level < 21 mol/l at the time of consent or in the past. ^4^Hemoglobin normal range: 12.0-15.5 g/L for females and 14-18 g/L in males. ^5^Normal range for ALT: 4-50 U/L; AST: 14-41 For the Chi-square test (Fisher’s exact test), there was 1 degree of freedom, and the effect size (Phi) was 0.058 ALT: alanine aminotransferase; AST: aspartate aminotransferase

Group/subgroup	Patients (n = 44)	Controls (n = 11)	Statistics
Age (years)			p = 0.43, t-test
Range	21-74	22-66	
Median	43	49	
Gender, n (%)			p = 0.12, Chi-square test
Female	31 (70%)	5 (45%)	
Male	13 (30%)	6 (55%)	
Low stature^1^, n (%)	22 (50%)		
Creatinine kinase (CK)^2^, n (%)			
Low	18 (42%)		
High	1 (2%)		
Vitamin D deficiency^3^, n (%)	11 (25%)		
Anemia, n (%)	2 (4.5%)		
Polycythemia^4^, n (%)	10 (23%)		
ALT/AST^5^, n (%)			
Low or high	5 (11%)		
Pain, n (%)			
Moderate to severe	29 (66%)		
Fatigue, n (%)			
Severe	30 (68%)		

Correlation between NF1 patient fatigue and pain

**Figure 2:**
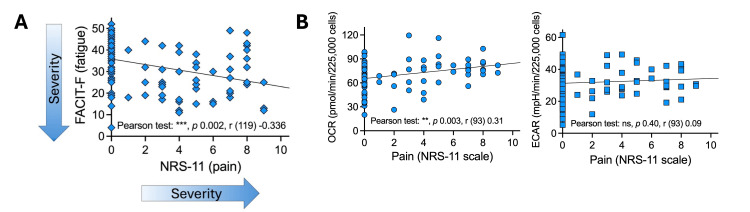
Assessing NF1 patient clinical phenotypes and mitochondrial function ^*^p < 0.05; ^**^p < 0.01; ^***^p < 0.005 (A) Correlation between severity of fatigue and pain for patients. Patient-reported fatigue (FACIT-F) and pain (NRS-11) were plotted and subjected to Pearson correlation analysis, indicating a significant negative relationship between responses (r(119) -0.34, p < 0.002). Of note, lower FACIT-F scores correspond to higher perceptions of fatigue, whereas higher NRS-11 scores correspond to higher perceptions of pain. (B) Correlation between OCR and pain (NRS-11 scale) on the left was significant (p 0.0003), but not that for ECAR on the right NF1: neurofibromatosis type 1; FACIT-F: the Functional Assessment of Chronic Illness Therapy-Fatigue; NRS: Numerical Pain Rating Scale; OCR: oxygen consumption rate; ECAR: extracellular acidification rate

Pain and fatigue were commonly reported among NF1 patients. The intensity of pain and fatigue in each patient was assessed using the NRS-11 and FACIT-F scales, respectively, at the time of blood collection. During the study, severe pain was reported by 15 (34%) patients at least once. Conversely, 15 (34%) patients reported low or mild pain. Regarding fatigue, 68% of patients reported experiencing severe fatigue at least once during the study. The scoring system for pain assigns higher values to greater severity, whereas for fatigue, higher values indicate lower severity. When patient-reported values were analyzed using a Pearson correlation test, there was a significant but low negative correlation between NRS-11 scores and FACIT-F scores (r(119) = -0.34, p < 0.002), as shown in Figure 2. This finding indicates that higher pain severity is associated with greater fatigue severity.

High metabolic and glycolytic bias in mitochondrial function for NF1 patients**

**Figure 3:**
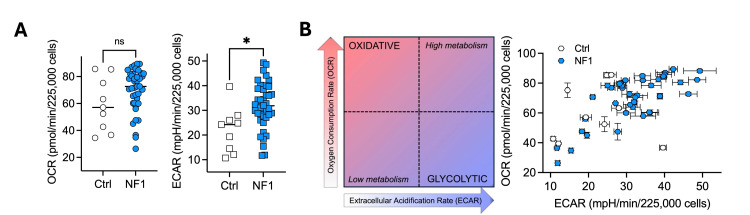
Examining the nature of mitochondrial function for NF1 patients ^*^p < 0.05; ^**^p < 0.01; ^***^p < 0.005 (A) Different mitochondrial function between controls and NF1 patients. Agilent Seahorse XF analyses of subject PBMCs showed a significant difference in ECAR between patients and those lacking NF1 defects (p = 0.015), but not for OCR (p = 0.16) using Welch’s t-test. (B) High metabolic and glycolytic bias in mitochondrial function for NF1 patients. A plot of OCR versus ECAR provides insights into (1) the magnitude of metabolism from low to high and (2) bias toward oxidative or glycolytic metabolism. When applied to subjects, NF1 patients demonstrated high rates of metabolism with a glycolytic bias NF1: neurofibromatosis type 1; PBMCs: peripheral blood mononuclear cells; OCR: oxygen consumption rate; ECAR: extracellular acidification rate

**Figure 4:**
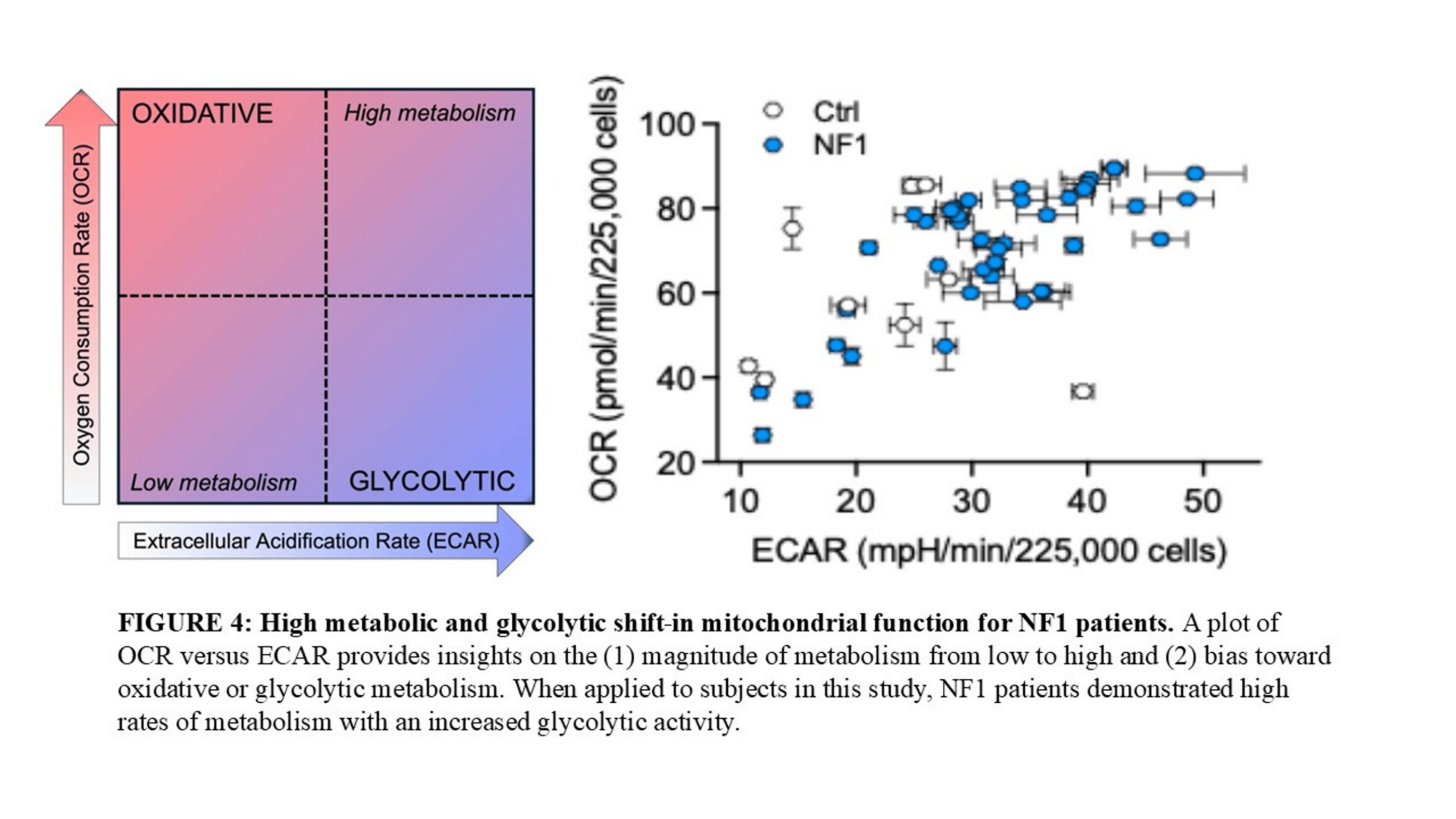
High metabolic and glycolytic shift in mitochondrial function for NF1 patients NF1: neurofibromatosis type 1; OCR: oxygen consumption rate; ECAR: extracellular acidification rate

Seahorse analyses of subject PBMCs yielded ECAR and OCR as measures of mitochondrial function. While there were single data points for each control, NF1 patients were sampled during three clinical visits and so provided two to three usable samples for analyses. The respective ECAR and OCR values demonstrated no statistically significant difference over the timing of those visits (data not shown). Figure 3 shows comparisons of Seahorse data between controls and NF1 patients. Based on a Welch’s t-test, ECAR was statistically higher for NF1 patients and those lacking NF1 defects (p = 0.015), but not for OCR (p = 0.16), despite a similar trend. A plot of OCR versus ECAR provides insights into (1) the magnitude of metabolism from low to high and (2) bias toward oxidative or glycolytic metabolism (Figure 4). This analysis showed that NF1 patients demonstrate high rates of metabolism with an elevated glycolytic activity, as indicated in Figure 4.

Correlations only between mitochondrial function and patient-reported pain

**Figure 5:**
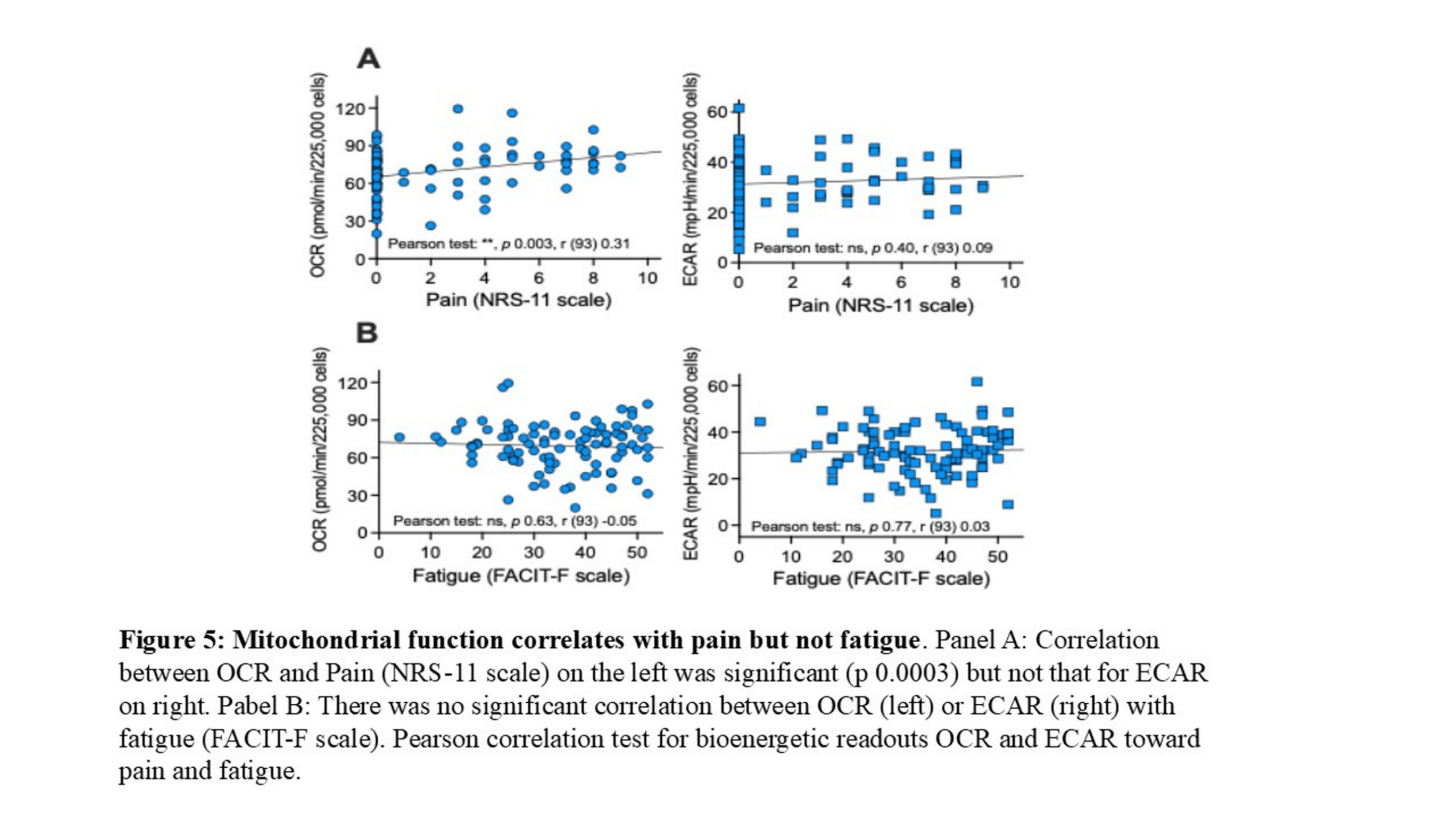
Mitochondrial function correlates with pain but not fatigue FACIT-F: the Functional Assessment of Chronic Illness Therapy-Fatigue; NRS: Numerical Pain Rating Scale; OCR: oxygen consumption rate; ECAR: extracellular acidification rate

Mitochondrial function can affect patients in many ways, so we assessed associations between serial ECAR and OCR values and the characteristics of NF1 patients, including clinical phenotypes for pain and fatigue measured on the same day as blood collection. From this analysis, there were no correlations with anthropometric measurements, such as height or weight (data not shown). This lack of correlation was also observed for analyses of mitochondrial readouts and laboratory results, including hemoglobin, vitamin levels, and liver function. Nevertheless, patient-reported pain, as measured by NRS-11 scores, showed a statistically significant but low correlation with OCR (r(93) = 0.31, p = 0.003, Pearson test); however, there was no correlation with ECAR (r(93) = 0.09, p = 0.4, Pearson test) (Figure 5, panel A). FACIT-F scores did not correlate with measured OCR (r(93) = -0.05, p = 0.63, Pearson test) or ECAR values (r(93) = 0.03, p = 0.77, Pearson test) (Figure 5, panel B).

Koselugo-treatment impact on mitochondrial function for NF1 patients

The study included five patients undergoing treatment with Koselugo (selumetinib), allowing exploration of the drug’s impact on patient mitochondrial function and outcomes. A comparison of ECAR and OCR values between untreated and treated NF1 patients did not reveal any statistically significant differences based on Welch’s t-test. Following treatment, three of the five patients exhibited adverse drug reactions, and therefore, they were withdrawn from that regimen. The limited sample size prevented statistical analyses.

## Discussion

In this pilot study, we first evaluated the feasibility of assessing bioenergetic markers of mitochondrial metabolism using PBMCs in patients with NF1. The use of PBMCs to detect metabolic stress in patients and serve as biomarkers for mitochondrial dysfunction is well-established for diseases such as diabetes, neurodegenerative disorders, cardiovascular disease, and various cancers [27-32]. PBMCs from patients with NF1 harbor the patients’ germline mutations and therefore reflect their impact on mitochondrial function [23-28]. After collecting blood samples from the subjects, we isolated PBMCs and measured mitochondrial function using an Agilent Seahorse to assess how patient characteristics influence bioenergetics for further analysis.

The Seahorse XF Analyzer provides real-time measurements of OCR and ECAR to assess mitochondrial function and glycolysis, respectively. Basal respiration refers to the cells’ baseline oxygen consumption under normal, unstressed conditions. It reflects the routine energy demands of the cell and the level of mitochondrial activity needed to meet these demands before any pharmacological intervention. While the primary focus is on OCR, ECAR is also measured as a secondary indicator. The ECAR signal arises from CO₂ release during the TCA cycle and glycolytic lactate production, offering insight into whether cells shift toward glycolysis in response to mitochondrial inhibition.

Although the patient population was small, it demonstrated many characteristic features of NF1, supporting the generalizability of the findings. Patients exhibited low stature, vitamin D deficiency, low CK levels, and high incidences of fatigue and pain. Moreover, we found that patients experiencing intense pain also suffered from severe fatigue.

Our bioenergetic measures provided a foundation for understanding how NF1 genetic lesions might impact mitochondrial function. These assays measured OCR as a proxy for oxidative phosphorylation and ECAR as an indicator of glycolysis, reflecting increased lactic acid export resulting from mitochondrial activity [33]. While oxygen consumption rates tended to be higher in NF1 patients in our study, they were not statistically different from those of controls. This observation contrasts with reports in NF1-deficient cells, which showed suppressed oxygen consumption rates compared with wild-type cells [13]. The discrepancies among these findings may be due to species differences, the nature of the genetic lesions, or the small number of controls. 

NF1 patient PBMCs are constitutionally heterozygous (NF1+/-) yet retain partial neurofibromin expression, whereas in vitro models typically involve complete NF1 ablation. The presence of residual neurofibromin activity in patients could explain differences in mitochondrial bioenergetics across studies and underscores the challenges of modeling the human condition. Subsequent studies have shown that the metabolic effects of neurofibromin loss are context-dependent, varying by cell type, the degree of NF1 inactivation, and microenvironment [34,35]. Our data suggest that in heterozygous NF1 PBMCs, glycolytic upregulation, indicated by increased ECAR, may occur before or independently of OCR suppression, consistent with a model in which Ras/ERK-mediated metabolic reprogramming enhances glycolysis before significantly affecting oxidative phosphorylation.

The elevated extracellular acidification rate observed in NF1 patients indicated a shift toward glycolytic metabolism, consistent with preclinical findings [13,36]. Glycolysis plays a critical role in fatty acid synthesis, suggesting that the mitochondrial effects of NF1 defects may contribute to lipid accumulation and elevated acetyl coenzyme A levels reported in NF1-deficient human and mouse cell lines, as well as in malignant peripheral nerve sheath tumors [36,37]. These features are characteristic of the Warburg effect, which is also observed in cancer cells [38,39]. Importantly, in this study, PBMCs from NF1 patients demonstrated compromised mitochondrial function, indicating that the germline defect may lead to widespread alterations in mitochondrial activity across all cells, potentially affecting their biological roles.

This pattern differs from that observed in NF1-null tumor cells, which exhibit a complete glycolytic switch, suggesting that partial neurofibromin expression is still retained in heterozygous PBMCs [13]. The substantial within-group variability in NF1 bioenergetic parameters may reflect the well-established clinical heterogeneity of NF1. Potential sources of metabolic variability include NF1 genotype, residual neurofibromin function, and differences in tumor burden. Additionally, disease severity, concomitant medications (including MEK inhibitors), and age could also contribute. Future studies with larger cohorts and detailed phenotypic characterization are needed to determine whether PBMC bioenergetic subgroups within the NF1 population represent clinically meaningful patient strata.

Systemic abnormal mitochondrial function in NF1 patients could compromise normal biological processes and thereby contribute to clinical outcomes. We explored this possibility by using bioenergetic measures to identify correlations with patient-reported pain and fatigue over time, with serial assessments conducted on the same day as PBMC collection. The majority of NF1 patients reported moderate to severe pain, and pain levels at the time of blood collection positively correlated with oxygen consumption rates. Increased oxygen consumption has been reported in patients with chronic pain from other conditions; however, research in this area is still preliminary and limited to a small number of studies [40,41].

Elevated oxygen consumption may lead to local oxygen depletion, potentially creating hypoxic conditions that contribute to pain, although further research is needed to validate this hypothesis. By contrast, neither mitochondrial measure correlated significantly with fatigue. The severity of patient-reported fatigue was positively correlated with pain, suggesting that the mechanisms underlying fatigue and pain may differ, with bioenergetics playing a role in pain but not fatigue. Another consideration is that the pain measurement tool (NRS-11) assesses pain at the moment the patient completes the questionnaire, whereas the fatigue tool (FACIT-F) evaluates overall fatigue over the preceding seven days rather than immediate fatigue levels.

Given altered mitochondrial function, NF1 treatments could modulate bioenergetics and thereby influence patient outcomes. MEK inhibitors are rapidly advancing in clinical use due to their efficacy in improving patient outcomes [14-16]. Nevertheless, this drug class is known to cause adverse side effects, possibly through the induction of mitochondrial dysfunction [17,18]. Such effects could exacerbate the already compromised mitochondrial function in NF1 patients. In our study, the number of serial samples from patients treated with MEK inhibitors was limited, so we could not draw definitive conclusions regarding extracellular acidification and oxygen consumption rates.

A limitation of our study is the small control group. As this was the first project to measure OCR and ECAR in PBMCs from this population, there was no prior data to determine the required sample size. Although the authors would have liked to increase numbers, funding constraints limited expansion. Minimal exclusion criteria for the control group were used to maximize its size and to closely reflect a population without NF1. The control group consisted of patients’ chaperones, typically spouses or adopted family members (not blood relatives), ensuring that environmental factors were similar to the NF1 group. The fact that we obtained statistically significant results with a small but diverse control group that approximated the patients’ age distribution is encouraging. The results of this study can now serve as a basis to calculate sample sizes for future, larger studies on energetic metabolism in NF1 patients.

## Conclusions

This pilot study provides the first characterization of peripheral blood mononuclear cell bioenergetics in NF1 patients using extracellular flux analysis. Extracellular acidification rates were significantly higher in NF1 patient PBMCs compared with controls (p = 0.015), indicating increased glycolytic activity. No significant difference in basal oxygen consumption rate was observed between the groups (p = 0.16). The association between OCR and patient-reported pain severity was a novel and unexpected finding, resulting in a significant correlation (p = 0.003), which may have implications for metabolic correlates of NF1-related pain if confirmed in future studies. These findings are hypothesis-generating and do not currently establish PBMC bioenergetics as a biomarker or therapeutic stratification tool.
